# Incidence of ideal candidates for focal therapy: A scoping study following the FALCON Consensus Statements

**DOI:** 10.1002/bco2.70121

**Published:** 2025-12-04

**Authors:** Jae Woong Jang, Nicole Handa, Ridwan Alam, Clayton Neill, Sai Kumar, Kent T. Perry, Edward M. Schaeffer, Hiten D. Patel, Ashley E. Ross

**Affiliations:** ^1^ Department of Urology Northwestern University Feinberg School of Medicine Chicago IL USA

**Keywords:** Focal Therapy, MRI, Prostate Cancer, Transperineal Biopsy

## Abstract

**Introduction:**

Ideal candidates for focal therapy (FT) for prostate cancer (PCa) have mpMRI‐visible ISUP Grade Group 2–3, localized disease. Transperineal (TP) prostate biopsies provide superior positional information of PCa in the transverse axis, and possibly higher PCa detection rates—important in choosing FT modalities. Here, we describe the frequency of optimal FT candidates among a large cohort of men with proven PCa identified from MRI‐guided targeted and systematic TP biopsy.

**Methods:**

We queried the Northwestern data warehouse for men with newly diagnosed PCa who had a positive mpMRI (PI‐RADS 3–5) and a TP biopsy prior to diagnosis from January 2018 to June 2024. Patients with disease optimal for FT were determined using modified FALCON consensus guidelines with emphasis on mpMRI and biopsy findings. We also explored the disparity in FT candidacy when using only target cores compared to combined target and systematic cores.

**Results:**

We identified 1342 men diagnosed with PCa on combined targeted and systematic TP biopsy after a positive mpMRI, of which 888 men had intermediate‐risk PCa. Of these 888 men, 439 patients (49.4%) had unilateral‐dominant disease, while 329 patients (37%) had unilateral‐dominant and anterior (106; 11.9%) or posterior‐dominant (223; 25.1%) disease. Up to half of the patients considered good candidates on target cores were considered FT‐ineligible on review of systematic cores.

**Conclusions:**

FT may be considered in up to 50% of patients with intermediate risk disease, while ideal candidates for functional preservation constitute a smaller number. Review of both targeted and systematic biopsy cores is crucial in determining FT candidacy.

## 1 INTRODUCTION

Focal therapy (FT) for prostate cancer (PCa) aims to selectively destroy PCa while maximizing sexual, urinary and bowel function, which are often compromised by radical treatment.^1^ Since the proposal of FT as a treatment modality for localized PCa, the implementation of FT has been cautious—owing partly to the need to confidently determine the localization and extent of disease on biopsy with high accuracy.^2^ Widespread usage of multiparametric MRI (mpMRI) to inform biopsies and the availability and reimbursement for ablative procedures have allowed for significant strides in FT implementation; however, the incidence and definition of appropriate candidates for FT remain unclear.^3^, ^4^

The most recent project aiming to establish a comprehensive list of consensus statements regarding FT is FALCON (FocAL therapy CONsensus).^5^ According to these statements, ideal candidates for FT have mpMRI‐visible Gleason Grade Group (GG) 2–3 disease that is localized. While the primary lesion(s) should also be visible on high‐quality mpMRI, the presence of disease outside of target cores does not contraindicate FT. The mpMRI must be followed by a combined targeted and systematic biopsy.

While previous studies have quantified the number of patients with unilateral localized PCa, such studies did not explore these numbers based on the current FALCON criteria or define FT candidates based on disease localization in the transverse plane (anterior vs. posterior), which may impact the choice of focal treatment and impact functional outcomes.^6^, ^7^, ^8^, ^9^, ^10^ Here, we sought to determine the incidence of optimal candidates for FT following the FALCON consensus guidelines while localizing PCa laterality and anterior versus posterior position in the prostate.

## 2 MATERIALS AND METHODS

### 2.1 Study Population

We queried the Northwestern data Warehouse involving 11 hospital systems for all patients between January 1, 2018 and March 1, 2025, with newly diagnosed PCa found on combined targeted and systematic transperineal (TP) biopsy preceded by an mpMRI. Patients undergoing TP biopsy were evaluated to allow for differentiation of anterior or posterior disease.^11^, ^12^, ^13^ Patients with TP biopsy performed within 6 months of mpMRI were selected for maximal correlation between MRI‐visible lesions (Prostate Imaging Reporting and Data System [PI‐RADS] score of 3–5) and biopsy target cores. Patients with a negative MRI—defined as PI‐RADS 2 or less—or an MRI report with limited information about the location of each lesion were excluded, as were men with a high likelihood of extracapsular extension on baseline mpMRI or evidence of metastatic disease within 3 months of diagnostic biopsy.

### 2.2 Study Design

We identified patients with optimal characteristics for FT for PCa among the total cohort. These characteristics were defined based on modified FALCON consensus guidelines with emphasis on mpMRI and biopsy findings. The FALCON consensus guidelines were “modified” in the sense that, among the numerous consensus statements, only the following criteria were considered for eligibility:

1. MRI‐visibility—defined as PI‐RADS scores of 3–5—of the biopsy‐identified primary lesion(s).
2. Intermediate risk disease—defined as GG 2–3.
3. Localized disease—defined as one of the following parameters:
  - Unilateral (“True‐Unilateral”) or clinically unilateral (“Unilateral‐Dominant”) disease, with Unilateral‐Dominant disease being defined as intermediate risk disease (GG2–3) on one side with contralateral low‐risk disease (GG1) or no cancer.
  - Transverse Axis:
    - Anterior (“True‐Anterior”) or clinically anterior (“Anterior‐Dominant”) disease, with Anterior‐Dominant disease being defined as intermediate risk disease (GG2–3) in the anterior prostate with low‐risk disease or no cancer in the posterior prostate.
    - Posterior (“True‐Posterior”) or clinically posterior (“Posterior‐Dominant”) disease, with Posterior‐Dominant disease being defined as intermediate risk disease (GG2–3) in the posterior prostate with low‐risk disease or no cancer in the anterior prostate.

The location of each MRI‐visible lesion was obtained from the mpMRI report. Each MRI‐visible lesion was correlated to appropriate target biopsy cores and analysed with the location of each positive systematic core to determine disease localization. Patients were required to have a Gleason score of 3 + 4 or 4 + 3 in a targeted biopsy core to be eligible for FT. Those with low‐risk PCa and those with foci of Gleason score 4 + 3 or 3 + 4 identified in only non‐target, systematic biopsy cores were considered ineligible for FT. We additionally conducted an analysis to compare the incidence of FT candidates based on 1) target biopsy cores only and 2) target + systematic cores.^7^, ^14^

This study complies with the Declaration of Helsinki and was approved by the Institutional Review Board.

## 3 RESULTS

**FIGURE 1:**
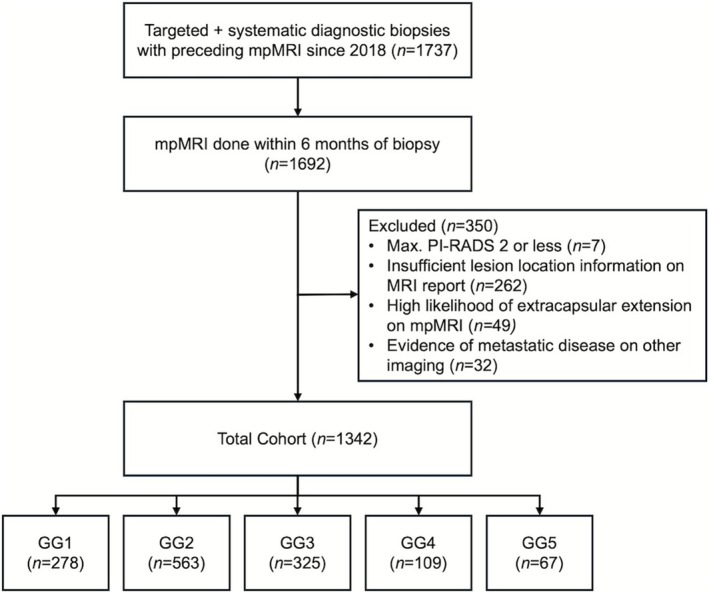
Consort diagram of query result to identification of total cohort with Gleason grade group distribution. mpMRI = Multiparametric magnetic resonance imaging. GG = Gleason grade group.

There were 1342 patients with a positive mpMRI (PI‐RADS 3–5) followed by a combined targeted and systematic diagnostic biopsy that met inclusion and exclusion criteria (Figure 1). Overall, the total cohort had a median age of 68 (IQR: 61.25–73) with a median PSA of 5.41 ng/ml (IQR: 4.11–7.98) and a median PSA density of 0.13 ng/ml/cc (IQR: 0.09–0.2). On mpMRI, most patients had a maximum PI‐RADS score of 4 (816/1324; 60.8%), followed by a score of 5 (350/1423; 26.1%). A total of 853 patients (63.6%) had a single lesion identified on mpMRI (Table 1).

**TABLE 1:** Demographic and clinical characteristics of 1342 patients with mpMRI (PI‐RADS 3–5), proven prostate cancer (any ISUP grade group) by combined MRI‐targeted and systematic transperineal prostate biopsy, and no evidence of metastatic disease.

Variable	n or median	% or IQR
**Age (yr)**	68	61.25–73
**Race**		
Black or African American	210	15.6%
White	934	69.6%
Other	30	2.2%
Unknown	168	12.5%
**Ethnicity**		
Hispanic or Latino/a	54	4%
Not Hispanic or Latino/a	1150	85.7%
Unknown	138	10.3%
**PSA (ng/mL)**	5.41	4.11–7.98
**PSA density (ng/mL/cc)**	0.13	0.09–0.2
**Prostate volume (cc)**	42	31–59
**Max. PI‐RADS**		
III	176	13.1%
IV	816	60.8%
V	350	26.1%
**Number of lesions**		
1	853	63.6%
2	417	31.1%
≥ 3	72	5.4%
**Max. GG on biopsy**		
1	278	20.7%
2	563	42%
3	325	24.2%
4	109	8.1%
5	67	5%

mpMRI = multiparametric magnetic resonance imaging. GG = grade group. PI‐RADS = Prostate Imaging Reporting and Data System.

Sixty‐four percent of men with mpMRI‐guided biopsy (888 patients) had intermediate‐risk disease (GG2–3) (Table 1). Disease location is summarized in Table 2. Among our cohort of men with intermediate‐risk, MRI‐visible disease, 49.4% were Unilateral‐Dominant and 32.5% had True‐Unilateral disease. Posterior‐Dominant disease was found in 34.6%, with 24.4% having True‐Posterior disease. Only 15% had MRI‐visible Anterior‐Dominant disease, and 11% had True‐Anterior disease. Combining these characteristics, the broad definition of localized disease included 11.9% of intermediate‐risk patients with Unilateral‐Dominant, Anterior‐Dominant disease and 25.1% with Unilateral‐Dominant, Posterior‐Dominant disease. Using narrow definitions, 6.9% of intermediate‐risk patients had True‐Unilateral, True‐Anterior disease and 14.2% of patients had True‐Unilateral and True‐Posterior disease.

**TABLE 2:** Incidence of patients with optimal characteristics for focal therapy candidacy.

Description	n	% (of total population)	% (of Max. GG2 or GG3)
**Total Cohort**	1342	100%	
**Max. GG 2 or GG 3**	888	66.2%	100%
**Unilateral‐Dominant** ^**a**^	439	32.7%	49.4%
**True‐Unilateral** ^**a**^	289	21.5%	32.5%
**Anterior‐Dominant** ^**a**^	133	9.9%	15%
**True‐Anterior** ^**a**^	98	7.3%	11%
**Posterior‐Dominant** ^**a**^	307	22.9%	34.6%
**True‐Posterior** ^**a**^	217	16.2%	24.4%
**Localized disease (Broad Definition)** ^**a**^	329	24.5%	37%
**Unilateral‐Dominant + Anterior‐Dominant** ^**a**^	106	7.9%	11.9%
**Unilateral‐Dominant + Posterior‐Dominant** ^**a**^	223	16.6%	25.1%
**Localized disease (Narrow Definition)** ^**a**^	187	13.9%	21.1%
**True‐Unilateral + True‐Anterior** ^**a**^	61	4.5%	6.9%
**True‐Unilateral + True‐Posterior** ^**a**^	126	9.4%	14.2%

^a^ All patients in each sub‐category had MRI‐visible (PI‐RADS 3–5) lesion(s) with maximum Gleason grade group of 2 or 3.

**FIGURE 2:**
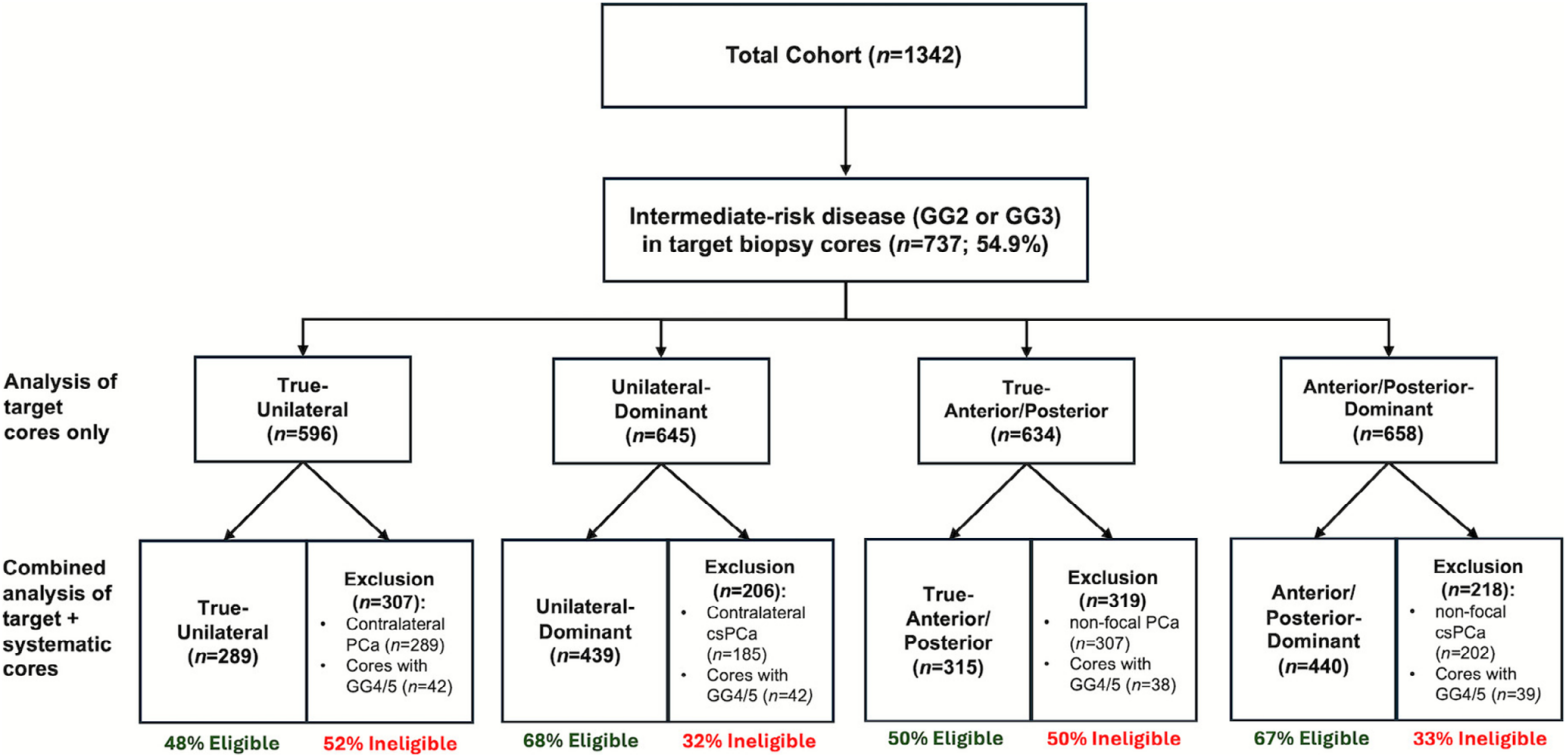
Consort diagram of patients with optimal characteristics for focal therapy based on target cores only vs. combined targeted and systematic cores.GG = Gleason grade group. PCa = Prostate cancer (any GG). csPCa = clinically significant prostate cancer (GG ≥ 2).

In our comparison of target cores only versus target and systematic cores in determining FT candidacy, we found that 54.9% (737/1342) of the total cohort had intermediate‐risk disease on target biopsy cores. Based purely on target cores, over 80% of these patients were deemed to have True‐Unilateral, Unilateral‐Dominant, True‐Anterior/Posterior or Anterior/Posterior‐Dominant disease (Figure 2). When adding information from systematic cores, as high as 52% (True‐Unilateral group; Figure 2) of the patients considered to be FT‐eligible on target core analysis were no longer eligible. A further breakdown of disease location and presumptive FT eligibility is shown in Figure 2.

## 4 DISCUSSION

In our cohort of over 1300 individuals diagnosed with non‐metastatic PCa on mpMRI‐informed TP prostate biopsy, up to roughly 25% were eligible for FT based on FALCON guidelines.^5^ Narrowing this to men with unilateral/anterior‐dominant, or true unilateral/anterior disease (men with likely best functional preservation after treatment) reduced FT candidates to 7.9% and 4.5%, respectively. Utilization of both systematic and targeted cores to define patients for FT was confirmed to be essential, with results of systematic biopsy cores conferring ineligibility in up to about 50% of potential FT candidates.

Ideal candidates for FT are those individuals where cancer control is necessary and can be achieved with limited to no functional morbidity^15^ Given the posterior location of the neurovascular bundles, this would be primarily anterior disease, preferably located on one lobe of the prostate. We found Anterior‐Dominant disease in just under 10% of our cohort, which is slightly lower than previously published literature—which may be due to limiting our series to TP biopsy and MRI targeting, allowing for improved prostate sampling.^16^, ^17^ We focused on TP biopsies in our series as biopsy templates provide locational information in the anterior and posterior plane. Beyond reduced infection rates, no need for antibiotics and a potential for improved prostate sampling in the anterior or apical prostate,^18^ TP biopsy may be ideal in qualifying men for FT as many FT modalities are delivered via a TP approach (i.e. cryoablation, irreversible electroporation, focal laser therapy).^8^, ^9^, ^19^, ^20^

In our analysis comparing target biopsies and systematic biopsies in determining FT candidacy, over half of those considered to be potential FT candidates were deemed ineligible, many having high grade disease discovered only on systematic biopsy. These results affirm the FALCON consensus recommendation that a high‐quality positive mpMRI followed by ≥ 3 targeted and ≥ 10 systematic biopsy cores are the key basis of determining FT eligibility.^5^ Our data are additionally in line with other studies favouring the use of systematic and targeted biopsies for superior sampling. For example, Ahdoot et al. (2020) compared the performance of targeted, systematic and combined targeted and systematic biopsies in men with suspicious lesions on mpMRI and found that the combined strategy led to the lowest rate of pathologic upgrading on radical prostatectomy.^14^ Further, Okabe et al. (2022) reported that 35.8% of patients with unifocal (unilateral) cancer based on targeted biopsy cores harboured contralateral cancer on systematic biopsy cores, illustrating the need for holistic review of targeted and systematic biopsy cores in determining the accurate extent of PCa.^7^

According to a recent meta‐analysis, FT has an oncologic failure (detection of ≥GG2 cancer in or out‐of‐field) rate of 14.4%.^21^ Thoughtful and evidence‐based selection of FT candidates is a key driver in FT treatment success. Current agreements on pathologic grading criteria for optimal FT candidacy is localized GG2 or GG3 disease, with localized GG1 disease patients being first recommended for active surveillance and those with > GG3 disease being excluded from FT consideration regardless of localization due to the potential of extra‐prostatic disease.^5^ Of note, while the 2017 Delphi consensus project reported that Gleason Score 3 + 3 disease at a single core (up to 1 mm) is acceptable in the untreated area, no such agreements were reached on the FALCON consensus project.^4^, ^5^ Thus, our analysis reported both the presence of out‐of‐field GG1 disease (Unilateral‐Dominant or Anterior/Posterior‐Dominant) or lack thereof (True‐Unilateral or True‐Anterior/Posterior) to reflect the current ambiguity revolving this topic. Additionally, the role of PSMA PET/CT in conjunction with traditional diagnostic tools is yet unclear especially in the evaluation of FT candidacy, with the only current agreement being that PSMA PET/CT is not a suitable replacement for MRI.^5^ However, with recent attention on MRI‐invisible but PET‐avid clinically significant PCa^22^ along with recent retrospective evidence suggesting that PSMA PET/CT in conjunction with MRI and systematic biopsy may improve the detection of clinically significant PCa in those with intermediate risk PCa,^23^ future efforts should further evaluate whether PSMA PET/CT should become a routine staging procedure for those with intermediate risk PCa, considering FT. Overall, current recommendations are subject to change with the results of ongoing and future studies, as suggested by FT being increasingly used for clinically significant disease (GG2–5) in clinical trials and observational studies across the years.^8^

There are several key limitations to keep in mind for this project. First, this was a retrospective scoping study with no centralized re‐review of mpMRI. Second, we did not limit our study to men undergoing prostatectomy where whole‐mount histopathological analysis could be performed. Next, not all eligibility criteria for FT candidacy were considered, with specific focus on mpMRI and biopsy requirements that pertained to the FALCON consensus statements. Characteristics not considered for FT eligibility in this paper that may still be clinically relevant for determining optimal FT candidacy include BRCA germline mutations, life expectancy and prostate volume. Thus, while the number of optimal candidates for FT is likely smaller than our estimates, FT may be at least considered in the identified patients based on their work‐up and localization of disease.

## 5 CONCLUSION

Approximately a quarter of all men diagnosed with localized PCa are candidates for FT, with roughly 5% being ideal candidates for therapy. mpMRI followed by systematic and targeted biopsies is critical in qualifying men for FT.

## AUTHOR CONTRIBUTIONS

JJ: Conceptualization, Methodology, Investigation, Writing – Original Draft. CN: Data Curation, Investigation. NH: Writing – Original Draft. KTP, EMS, HDP, AER: Supervision, Writing – Review and Editing. AER: Conceptualization, Supervision.

## CONFLICT OF INTEREST STATEMENT

Dr. Edward M. Schaeffer is a consultant and/or speaker for the following: Astellas Pharmaceuticals Global Development Inc, Pfizer International Inc.

Dr. Ashley E. Ross is a consultant and/or speaker for the following: Astellas Pharmaceuticals Global Development Inc, Astrazeneca, Bayer HealthCare Pharmaceuticals Inc, BillionToOne Inc, Janssen Pharmaceuticals Inc, Sumitomo Pharmaceuticals America Inc, Lantheus Medical Imaging Inc, Pfizer International Inc, Veracyte Inc.

All other authors declare that there is no conflict of interest regarding the publication of this article.

## FUNDING STATEMENT(S)

Dr. Hiten D. Patel is supported by a Prostate Cancer Foundation Young Investigator Award (24YOUN22) and a Developmental Research Program grant from the Polsky Urologic Cancer Institute and SPORE in Prostate Cancer at the Robert H. Lurie Comprehensive Cancer Center (P50CA180995).

No other funding was received for this research.
